# Lower levels of FOXP3 are associated with prolonged inflammatory responses in kidney transplant recipients

**DOI:** 10.3389/fimmu.2023.1252857

**Published:** 2023-09-13

**Authors:** Qais W. Saleh, Afsaneh Mohammadnejad, Martin Tepel

**Affiliations:** ^1^ Department of Nephrology, Odense University Hospital, Odense, Denmark; ^2^ Cardiovascular and Renal Research, Department of Molecular Medicine, University of Southern Denmark, Odense, Denmark; ^3^ Epidemiology, Biostatistics and Biodemography, Department of Public Health, University of Southern Denmark, Odense, Denmark

**Keywords:** kidney transplantation, FOXP3, inflammation, T regulatory cells, C reactive protein, RNA transcripts, RNA splice variants

## Abstract

**Background:**

Immunosuppressive treatment of kidney transplant recipients is mainly aimed at pro-inflammatory T effector cells, yet they also target the immunosuppressive T regulatory cells. Here, we test the hypothesis that low levels of the master gene regulator of T regulatory cells, forkhead box P3 (FOXP3) splice variants, are associated with prolonged inflammatory responses to stimuli.

**Methods:**

From blood samples obtained the first – and 29^th^ day post-transplant, we extracted peripheral blood mononuclear cells and measured mRNA levels of Total FOXP3, pre-mature RNA FOXP3 (pre-mRNA FOXP3), full length FOXP3 (FOXP3fl) and, FOXP3 splice variant excluding exon two (FOXP3d2). We defined the primary outcome as the number of days in which C reactive protein (CRP) was above 50 mg/L. CRP levels were gathered in two periods, the first from the second to 29 days post-transplant, and the second from 30 to 57 days post-transplant. The association was tested using adjusted negative binomial regression.

**Results:**

From 507 included kidney transplant recipients, 382 recipients had at least one CRP measurement >50 mg/L in the first period, median duration of elevated CRP was 4 days [interquartile range (IQR) 2 to 6]. In the second period, 69 recipients had at least one CRP measurement >50 mg/L, median duration of elevated CRP was 3 days [IQR 2 to 5]. In the first period, we found a significant association between lower levels of Total FOXP3 and prolonged duration of CRP elevation, incidence rate ratio 0.61 (95% confidence interval 0.46-0.80), p<0.01.

**Conclusion:**

Lower levels of total FOXP3 mRNA levels in peripheral blood of kidney transplant recipients are associated with prolonged duration of inflammatory responses regardless of the underlying stimuli.

## 1 Introduction

Kidney transplant recipients are treated with immunosuppressive medicines to prevent rejection of the transplanted kidney allograft. Immunosuppressive medicines mainly target and weaken the adaptive immune system (1), which may result in increased risk of severe and prolonged inflammatory responses to inflammatory stimuli. Severe and prolonged inflammatory responses aggravate recipient morbidity and may cause graft loss or mortality (2).

Immunosuppressive medicines also target T regulatory cells (3–9), which regulate and limit immune responses to inflammatory stimuli (10). The effect of immunosuppressive medicines may be reflected by changes in T regulatory cell forkhead box P3 (FOXP3) mRNA splice variant levels, as FOXP3 mRNA levels may decrease indirectly by depletion or inhibition of T regulatory cells in response to immunosuppressive therapy (3–5, 7). Therefore, FOXP3 mRNA splice variant levels may help in identifying kidney transplant recipients that may develop severe and prolonged inflammatory responses. Furthermore, the effects of immunosuppressive therapy on T regulatory cells may compromise the anti-inflammatory function of T-regulatory cells, and lead to pro-inflammatory conditions and a tendency for more severe and prolonged inflammatory responses.

C-reactive protein (CRP) is a well-established and widely used marker of inflammation. CRP is produced in the liver and is released mainly in response to stimulation by interleukin 6 (IL-6). Following stimulation, CRP levels rise within 24 to 72 hours, and stay elevated until the inflammatory stimulus ends, thereafter CRP levels decrease exponentially over 18-20 hours (11). Prolonged and higher levels of CRP are associated with severe inflammatory conditions such as bacterial infections, acute rheumatological disorders and SARS-COV-2 infections (12–14). Thus, prolonged, and very high CRP measurements reflect the severity of inflammatory episodes.

In this study, we hypothesized that low FOXP3 mRNA splice variant levels are associated with a higher tendency for prolonged and severe inflammatory responses in kidney transplant recipients. We aimed to investigate this association in a cohort of incident kidney transplant recipients.

## 2 Methods

### 2.1 Study design, clinical data, sample collection and ethical statements

This study is an accessory study of the ongoing, prospective, single-center project, molecular monitoring after kidney transplantation project (MoMoTx) (15). MoMoTx has screened incident kidney transplant recipients for inclusion since its inception in January 2011. Participants are considered eligible for enrollment if they are older than 18 years, are not recipients of multiple organ transplantation, and are able to provide informed consent. Recipients of second/more kidney re-transplantations are enrolled as new participants. MoMoTx screens eligible participants at Odense University Hospital.

**Figure 1:**
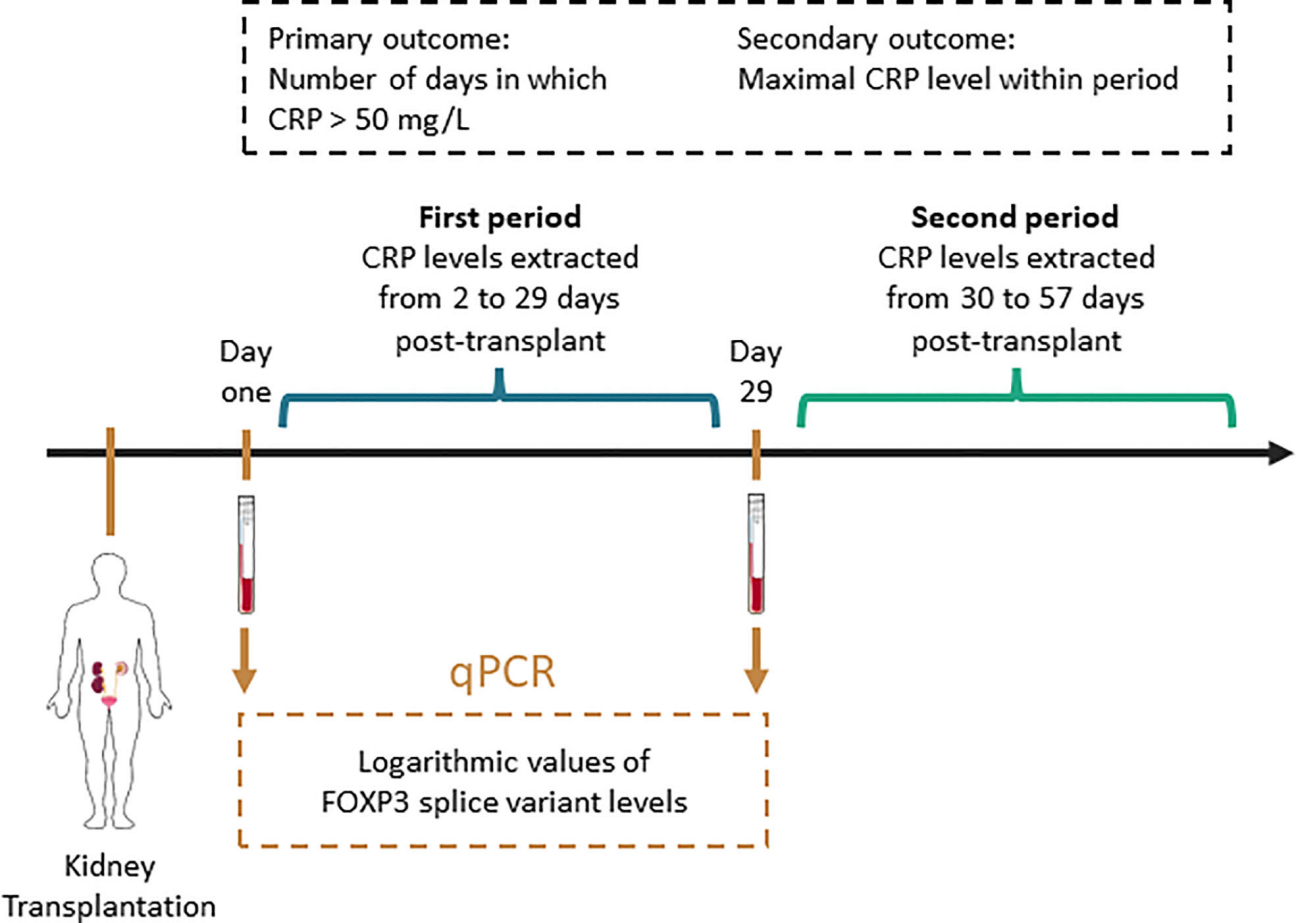
Schematic presentation of methodology. CRP, C reactive protein; qPCR, quantitative polymerase chain reaction. This figure contains modified art from Servier Medical Art, provided by Servier, licensed under a creative commons attribution 3.0 unported license.

In this study, we evaluated MoMoTx participants transplanted from January 2011 until August 10. 2021 for inclusion. Recipients who had available laboratory data for determination of the outcome variable were included in the final analysis. In pre-liminary data we observed that FOXP3 mRNA levels were lowest the first day post-transplant. Therefore, we used blood samples collected one day post-transplant. Because T regulatory cells, and consequently FOXP3 levels increase transiently after their initial fall (3, 4), we also used blood samples collected 29 days post-transplant. **Figure 1** illustrates the study design.

Recipient clinical data was gathered through review of electronic medical records, and included: recipient age; sex; anthropometric measurements; cause of end-stage kidney disease; clinical diagnoses including diabetes mellitus and coronary artery disease; active tobacco consumption; duration of dialysis (vintage) in months; type of dialysis (hemodialysis, peritoneal dialysis); number of transplantations; kidney donor type (ABO-compatible, ABO-incompatible living donor, deceased donor); total number of human leukocyte antigen (HLA) mismatches; delayed graft function defined as the need for renal replacement therapy within the first week post-transplant; immunosuppressive induction therapy such as IL2-receptor antibodies (Basiliximab), Anti-Thymocyte globulins (Thymoglobulin), corticosteroids and anti-CD20 antibodies (Rituximab); immunosuppressive maintenance therapy including tacrolimus, cyclosporine and mycophenolate acid. Recipient CRP laboratory values were extracted from the day of transplantation and until 60 days post-transplant. Donor data were collected through review of electronic medical records.

This study adheres to the ethical standards of the declaration of Helsinki and Istanbul, and has been granted approval of the local ethics committee (Den Videnskabsetiske Komite for Region Syddanmark, Project-ID: 20100098).

### 2.2 CRP as the outcome variable

To coincide with the two blood samples used to determine FOXP3 measurements, the primary- and secondary outcome variables were determined in two periods. The first period spanned from the second to 29^th^ day post-transplant. To match the duration of the first period, the second period spanned from the 30^th^ to 57^th^ day post-transplant. In each period, we defined the count of days post-transplant in which CRP levels are above 50 mg/L as the primary outcome. We chose the cut-off value of 50 mg/L to exclude episodes of limited or chronic inflammatory responses, which are associated with lower CRP level elevation (16–18). In recipients with at least one CRP measurement above 50 mg/L, the secondary outcome was defined as the maximal CRP level within each period (**Figure 1**).

### 2.3 FOXP3 mRNA splice variant measurements

We adapted previously described procedures by our group (15, 19, 20). In short, we used heparinized blood samples collected one – and 29 days post-transplant from included kidney transplant recipients. We then extracted peripheral blood mononuclear cells using density gradient centrifugation with Histopaque (Sigma-Aldrich, St. Louis, MO, USA; density 1.077 g/mL), and washed the cells with Hanks’ balanced salt solution (Thermo Fisher scientific, Waltham, MA, USA). These were then suspended in TRIzol (Invitrogen, Thermo Fisher Scientific, Waltham, MA, USA). We proceeded to extract total RNA using RNeasy Mini kit including RNase-free DNase set (Qiagen, Hilden, Germany), which was used for complementary DNA synthesis with QuantiTect Reverse Transcription kit (Qiagen). Finally, using SYBR green, we performed quantitative reverse transcription polymerase chain reaction with LightCycler 96 (Roche Diagnostics, Basel, Switzerland), annealing temperature was set to 63° C.

**Figure 2:**
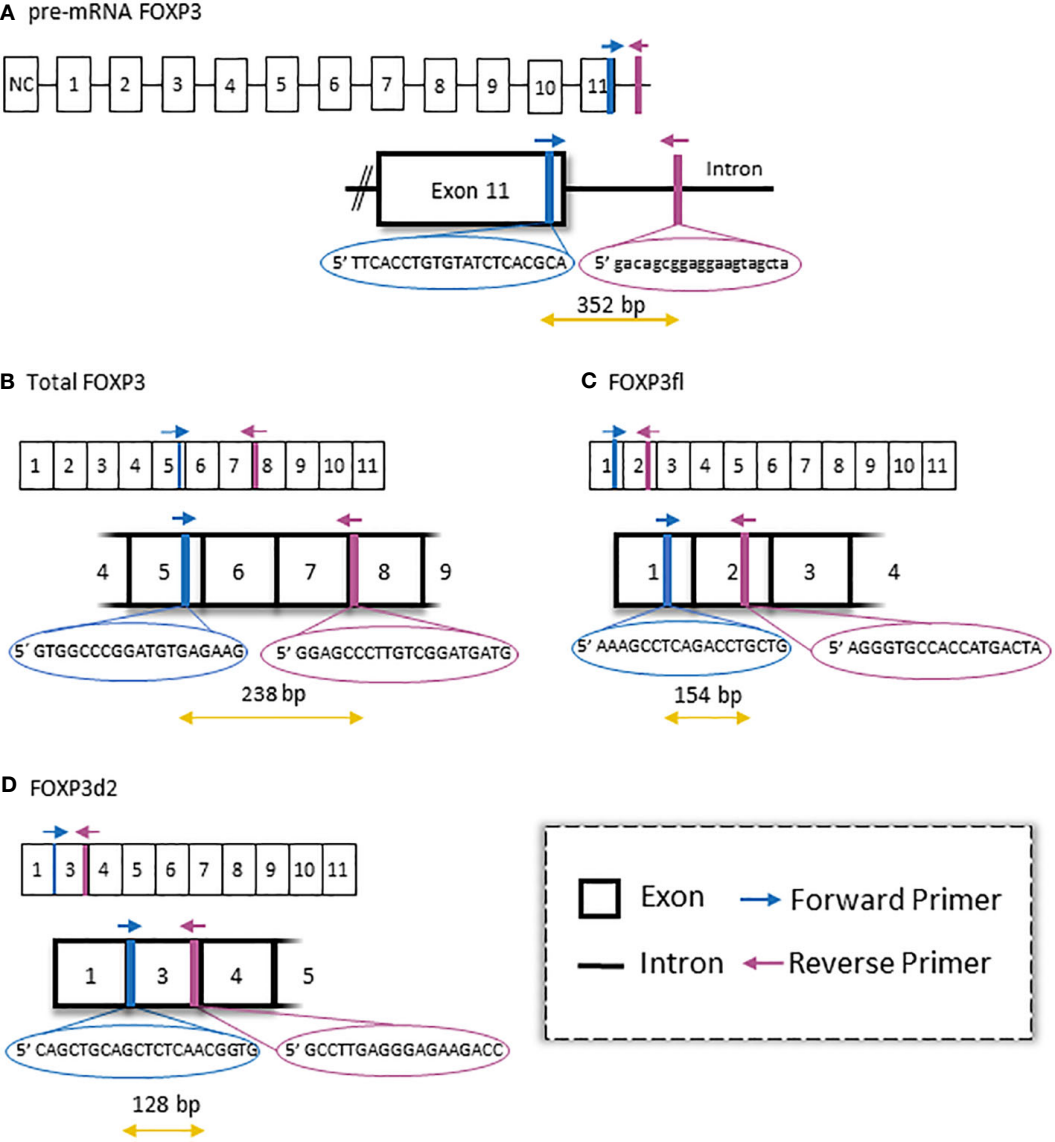
Illustration of forkhead box P3 (FOXP3) splice variants and primer pairs used to detect each variant in quantitative polymerase chain reaction. **(A)** pre-mature mRNA. **(B)** primers to detect both major mature FOXP3 splice variants. **(C)** full length FOXP3 (FOXP3fl) splice variant. **(D)** FOXP3 splice variant in which exon 2 is skipped (FOXP3d2).

We measured FOXP3 mRNA transcripts using four primer pairs (**Figure 2**). The following primer pairs were applied: total mature FOXP3 mRNA (Total FOXP3) forward 5’-GTGGCCCGGATGTGAGAAG-3’ and reverse 5’-GGAGCCCTTGTCGGATGATG-3’; pre-mature FOXP3 mRNA (pre-mRNA FOXP3) forward 5’-TTCACCTGTGTATCTCACGCA-3’ and reverse 5’-gacagcggaggaagtagcta-3’; full length mature FOXP3 mRNA (FOXP3fl) forward 5’-AAAGCCTCAGACCTGCTG-3’ and reverse 5’-AGGGTGCCACCATGACTA-3’; mature FOXP3 mRNA splice variants with exon two skipping (FOXP3d2) 5’-CAGCTGCAGCTCTCAACGGTG-3’ and reverse 5’-GCCTTGAGGGAGAAGACC-3’. We also measured β-actin with forward 5’-GGACTTCGAGCAAGAGATGG-3’ and reverse 5’-AGCACTGTGTTGGCGTACAG-3’ primers. Finally, we calculated normalized FOXP3 splice variants with the equation: Normalized ratio = ET^(CqR-CqT)^ with ET, efficiency of target amplification; CqT and CqR, quantification cycle at target/reference detection (19).

### 2.4 Statistical analysis

Continuous variables are presented as median [interquartile range] and categorical values are presented as number (percent). Normality of continuous variables were tested using QQ-plots, and tests of comparisons consisted of paired or unpaired t-test and Mann-Whitney U where appropriate. Categorical data were compared with Fishers Exact test or Chi-squared test where appropriate.

**Figure 3:**
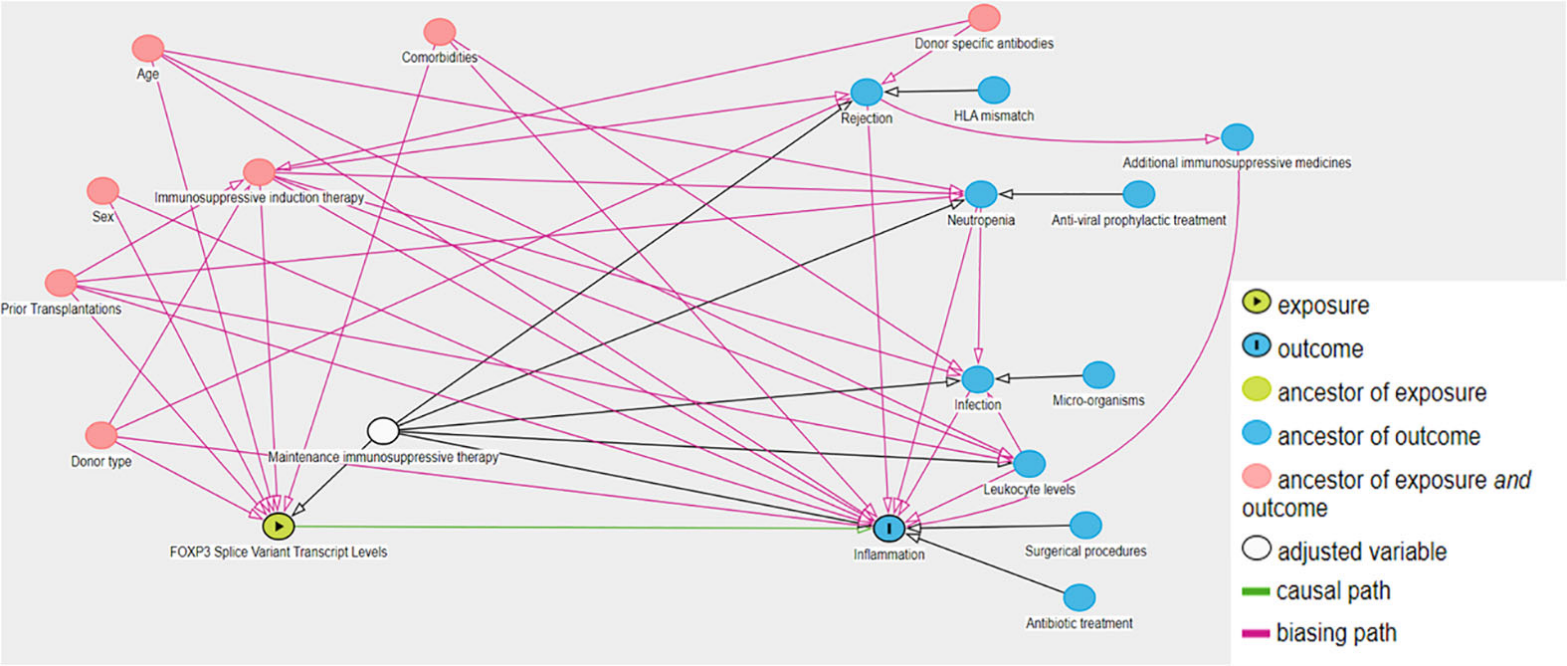
Directed acyclic graph of exposure variable of interest, outcome and associated variables. We used the backdoor pathway criteria to detect potential confounding factors, in which ancestors of the exposure and outcome variables were considered potential confounding factors and included in multivariable regression models. Maintenance therapy is adjusted by design as kidney transplant recipients receive tacrolimus and mycophenolate acid according to a defined protocol. The causal paths are based on literature.

To facilitate comprehension of FOXP3 mRNA levels, and to make results more comprehensive, FOXP3 mRNA levels were converted to a logarithmic scale. To test the association between the primary outcome and FOXP3 mRNA levels, we performed Negative Binomial regression analysis as overdispersion was observed in Poisson regression (21). The association between the secondary outcome and FOXP3 mRNA levels were tested using multivariable linear regression. Potential confounding factors were identified with directed acyclic graphs using the back-door pathway criteria (22, 23). Ancestors common to the exposure – and outcome variables were considered potential confounding factors and were included in the final models. Assumptions of associations between potential confounding factors, the outcome variable and/or the exposure variables were based on the literature. All assumptions, including citations, are detailed in **Supplemental Table 1**, and were visualized using DAGitty (www.dagitty.net/dags.html) (**Figure 3**). The co-factors describing comorbidities (Diabetes, cardiovascular disease, and Hypertension) were combined to the number of comorbidities to simplify the regression models. Immunosuppressive induction therapeutics (Basiliximab, corticosteroids, Thymoglobulin and Rituximab) were re-coded as combined regiments. We considered an association between an exposure variable of interest and the outcome variable to be statistically significant if the p-value was lower than 0.05.

To provide a better model fit in linear regression, the secondary outcome variables were converted to a logarithmic scale. Finally, we assessed the assumption of the linear regression,including linearity, homogeneity of variance and normality of residuals. All statistical analyses were performed in R (version 4.2.3, R Foundation for Statistical Computing, Vienna, Austria).

## 3 Results

### 3.1 Participant attributes

**Figure 4:**
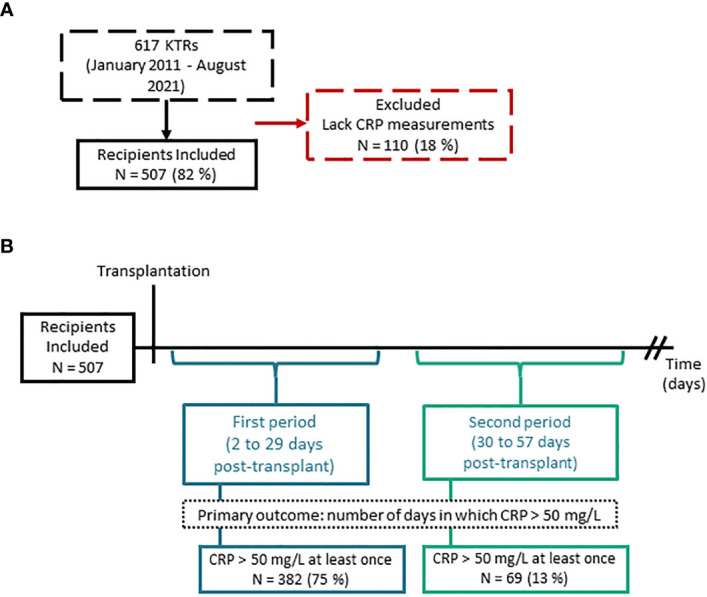
Flow diagram of study population and study findings. **(A)** Flow diagram of included and excluded kidney transplant recipients (KTRs). **(B)** Schematic of the study design with number of recipients which had at least one C reactive protein record exceeding 50 mg/L within the first period (2 to 29 days post-transplant) and second period (30 to 57 days post-transplant).

We assessed 617 kidney transplant recipients enrolled in MoMoTx for inclusion. Of the assessed participants, 507 had available CRP measurements within both periods, and were included in the final analysis (**Figure 4A**). As detailed in **Table 1**, the included participants had a median age of 53 [IQR 42 to 62] years and 342 (67%) were males. Kidney donations were obtained from deceased donors in 302 (60%) recipients, living ABO-compatible donors in 152 (30%), and living ABO-incompatible donors in 53 (10%). Only 65 (13%) recipients had been transplanted previously. Glomerulonephritis was the most common underlying cause of kidney disease and was present in 164 (32%) recipients. Maintenance immunosuppressive therapy consisted of tacrolimus and mycophenolate acid in all included recipients. Inductive immunosuppressive therapy was comprised of seven possible combinations of Basiliximab, Rituximab, Thymoglobulin or Corticosteroids. The most common immunosuppressive induction regimen was with Basiliximab only, which was administered to 367 (72.3%) recipients. In accessible donor data (**Supplemental Table 2**), the median age of kidney donors was 55 [IQR 46 to 64] years, 111 (45%) were male, and the median cold ischemic time was 780 [IQR 585 to 1050] minutes.

**Table 1:** Baseline characteristics of included kidney transplant recipients.

Variable	Statistic
Age (years)	53 [42 to 62]
Male sex, N (%)	342 (67%)
Height (cm)	175 [168 to 182]
Weight (kg)	82 [70 to 93]
Diabetes, N (%)	94 (19%)
Cardiovascular disease, N (%)	68 (13%)
Active tobacco use, N (%)	147 (29%)
Duration of dialysis (months)	12 [2 to 24]
Second/more transplantation, N (%)	65 (13%)
Number of HLA mismatches (range within 0-8)	3 [2 to 4]
Plasma creatinine pre-transplant (µmol/l)	710 [545 to 914]
Delayed graft function	62 (16%)
Experienced at least one rejection episode within 60 days post-transplant	43 (8.5%)
Cause of kidney disease, N (%) Glomerulonephritis Diabetic nephropathy Hypertensive nephropathy Hydronephrosis Cystic kidney disease Cancer Unknown	164 (32.34%) 76 (14.99%) 74 (14.59%) 23 (4.53%) 81 (15.97%) 6 (1.18%) 83 (16.37%)
Type of dialysis, N (%) Pre-emptive Hemodialysis Peritoneal dialysis	106 (21%) 272 (54%) 129 (25%)
Donor type, N (%) Deceased Living ABO-compatible Living ABO-incompatible	302 (60%) 152 (30%) 53 (10%)
Induction Therapy, N (%) Basiliximab Basiliximab and corticosteroids Basiliximab, corticosteroids and Rituximab Corticosteroids and Rituximab Thymoglobulin Thymoglobulin, corticosteroids Thymoglobulin, corticosteroids and Rituximab	367 (72.3%) 18 (3.6%) 35 (6.9%) 9 (1.8%) 2 (0.4%) 26 (5.1%) 50 (9.9%)
Maintenance therapy, N (%) Tacrolimus Mycophenolate Acid	507 (100%) 507 (100%)
Logarithmic values of FOXP3 levels first post-transplant day, N_samples_ = 470 Total FOXP3 Pre-mRNA FOXP3 FOXP3fl FOXP3d2	-3.70 [-3.99 to -3.40] -4.43 [-4.69 to -4.13] -4.02 [-4.34 to -3.76] -3.77 [-4.04 to -3.53]
Logarithmic values of FOXP3 levels 29 days post-transplant, N_samples_ = 426 Total FOXP3 Pre-mRNA FOXP3 FOXP3fl FOXP3d2	-3.59 [-3.91 to -3.34] -4.36 [-4.63 to -4.11] -3.89 [-4.22 to -3.64] -3.70 [-3.99 to -3.45]

Continuous values are reported as median [interquartile range], and categorical values are reported as number (percent). FOXP3, forkhead box P3; FOXP3fl, FOXP3 splice variant in which all exons are expressed; FOXP3d2, FOXP3 splice variant in which all exons but exon two are expressed; Total FOXP3, expression of both FOXP3fl and FOXP3d2 variants. Pre-mRNA: pre-mature RNA FOXP3 splice variants.

Total FOXP3 mRNA levels were more abundantly expressed than pre-mRNA FOXP3 levels when measured the first day post-transplant (logarithmic value -3.70 [IQR -3.99 to -3.40] vs -4.43 [IQR -4.69 to -4.13], p <0.01) and 29 days post-transplant (logarithmic value -3.59 [IQR -3.91 to -3.34] vs -4.36 [IQR -4.63 to -4.11], p <0.01). Similarly, FOXP3d2 mRNA levels were more abundantly expressed compared to FOXP3fl mRNA levels the first day post-transplant (logarithmic value -3.77 [IQR -4.04 to -3.53] vs -4.02 [IQR -4.34 to -3.76], p <0.01) and 29 days post-transplant (logarithmic value -3.70 [IQR -3.99 to -3.45] vs -3.89 [IQR -4.22 to -3.64], p <0.01). All FOXP3 mRNA measurements were lower at day one compared to day 29, Total FOXP3 (p<0.01), pre-mRNA FOXP3 (p=0.01), FOXP3fl (p<0.01), FOXP3d2 (p<0.01).

In *ad hoc* analysis, we explored changes in FOXP3 mRNA levels following transplantation. We measured pre-transplant FOXP3 levels in available blood samples from included patients (N = 248) and compared pre-transplant FOXP3 levels to FOXP3 levels measured at day one and day 29 post-transplant. Total FOXP3, pre-mRNA FOXP3, FOXP3fl and FOXP3d2 levels decreased significantly from pre-transplant levels to day one post-transplant, then increased significantly from day one to day 29 (**Supplemental Table 3**).

### 3.2 CRP values during the first – and second period

During the first period (2 to 29 days post-transplant), a CRP value above 50 mg/L was measured in 382 (75%) recipients at least once, and the median number of days in which CRP was above 50 mg/L was 4 [IQR 2 to 6] (**Table 2**, **Figure 4B**). The median maximal CRP value within this period was 122 mg/L [IQR 88 to 181]. During the second period (30 to 57 days post-transplant), 69 (13%) recipients had a measurement of CRP > 50 mg/L at least once (**Figure 4B**). The median number of days in which CRP was above 50 mg/L was 3 [IQR 2 to 5], and the median maximal CRP value was 117 mg/L [68 to 177] (**Table 2**).

**Table 2:** Descriptive statistics of primary outcome (number of days with CRP > 50 mg/L) and secondary outcome (maximal level of CRP within a period).

Outcome	Period	Number of recipients with positive outcome	Median [Interquartile range]
Number of days with CRP > 50 mg/L	2 to 29 days post-transplant	382 (75 %)	4 [2 to 6]
	30 to 57 days post-transplant	69 (13 %)	3 [2 to 5]
Maximal level of CRP (mg/L)	2 to 29 days post-transplant	382 (75 %)	122 [88 to 181]
	30 to 57 days post-transplant	69 (13 %)	117 [68 to 177]

### 3.3 Association of FOXP3 levels with duration of inflammatory responses

To test the association between FOXP3 mRNA levels and the primary outcome (count of days post-transplant in which CRP levels are above 50 mg/L), we performed negative binomial regression analysis. We included the following co-factors as potential confounding factors: recipient age, sex, prior kidney transplantation, donor type, type of induction therapy regiment, and number of comorbidities.

**Figure 5:**
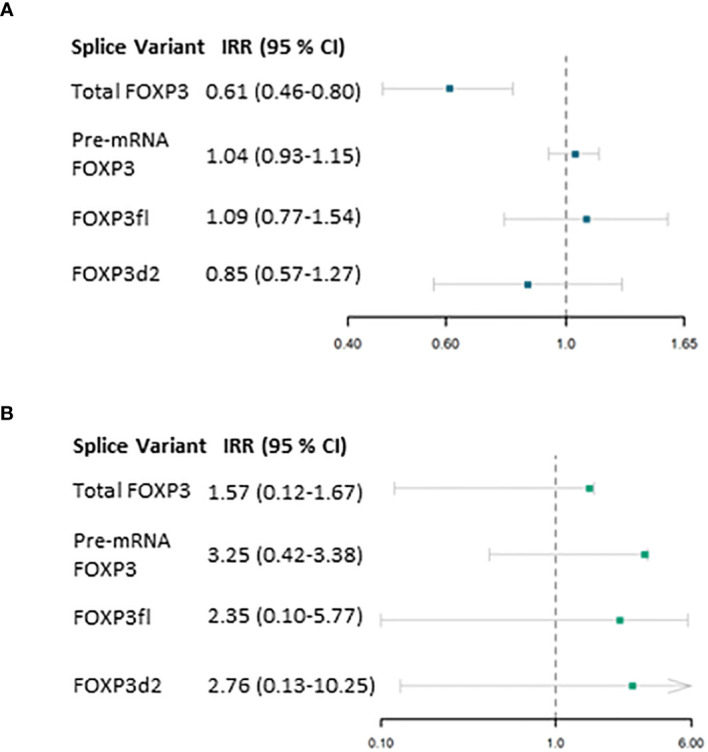
Results from Negative Binomial regression model. **(A)** association of forkhead box P3 (FOXP3) splice variants measured one day post-transplant and the primary outcome (number of days in which C reactive protein exceeds 50 mg/L) within the first period (2 to 29 days post-transplant) adjusted for confounding factors. **(B)** association of FOXP3 splice variants measured 29 days post-transplant and the primary outcome (number of days in which C reactive protein exceeds 50 mg/L) within the second period (30 to 57 days post-transplant). IRR, Incidence rate ratio; 95% CI, 95 % Confidence interval.

In the first period (**Table 3**), Total FOXP3 was significantly associated with the outcome variable (estimate (β) -0.49, p<0.01). We calculated the incidence ratio rate (IRR) by exponentiating the obtained estimate which yielded an IRR of 0.61, 95% CI 0.46-0.80 (**Figure 5A**).

**Table 3:** Results of Negative Binomial regression analysis, where the outcome variable is the number of days in which C reactive protein > 50 mg/L.

	Outcome within 2 to 29 days post-transplant (N = 507)		Outcome within 30 to 57 days post-transplant (N = 507)	
Variable	Estimate ±standard error	P-value	Estimate ±standard error	P-value
Total FOXP3	-0.49 ± 0.13	< 0.01	-0.79 ± 0.50	0.11
Pre-mRNA FOXP3	0.04 ± 0.05	0.45	0.16 ± 0.33	0.61
FOXP3fl	0.09 ±0.17	0.59	-1.5 ± 0.63	0.80
FOXP3d2	-0.15 ± 0.20	0.44	0.01 ± 0.83	0.98
Age	0.006 ± 0.003	0.06	0.02 ± 0.01	0.06
Male sex	-0.01 ± 0.09	0.90	-0.71 ± 0.41	0.08
Prior kidney transplantation	0.46 ± 0.14	< 0.01	1.48 ± 0.61	0.01
Number of comorbidities	0.17 ± 0.06	0.01	-0.49 ± 0.29	0.09
Donor type				
Deceased	Reference		Reference	
Living ABO-compatible	-0.29 ± 0.10	< 0.01	0.01 ± 0.47	0.96
Living ABO-incompatible	-0.35 ± 0.31	0.26	0.76 ± 1.30	0.55
Induction therapy				
Basiliximab	Reference		Reference	
Basiliximab and corticosteroids	-0.73 ± 0.24	< 0.01	-0.01 ± 0.47	0.15
Basiliximab, corticosteroids and rituximab	-1.34 ± 0.34	< 0.01	-7.64 ± 1.30	0.79
Corticosteroids and rituximab	-2.49 ± 0.70	< 0.01	-1.75 ± 1.24	0.99
Thymoglobulin	-1.83 ± 0.76	0.01	-3.69 ± 6711000	1.00
Thymoglobulin and corticosteroids	-2.84 ± 0.42	< 0.01	-2.01 ± 1.16	0.08
Thymoglobulin, corticosteroids and rituximab	-1.84 ± 0.25	< 0.01	-2.64 ± 1.04	0.01

FOXP3, forkhead box P3. FOXP3fl, FOXP3 splice variant in which all exons are expressed. FOXP3d2, FOXP3 splice variant in which all exons but exon two are expressed. Total FOXP3, expression of both FOXP3fl and FOXP3d2 variants. Pre-mRNA: pre-mature RNA FOXP3 splice variants.

Considering the remaining co-factors, prior kidney transplantation, number of comorbidities, donor type and induction therapy regimens were significantly associated with the outcome variable. Prior kidney transplantation was significantly associated with prolonged elevation of CRP levels (β 0.46, p < 0.01). Increasing number of comorbidities was also associated with prolonged elevation of CRP (β 0.17, p = 0.01). Living ABO-compatible kidney donation was associated with shorter duration of elevated CRP (β -0.29, p < 0.01), compare to deceased kidney donations as reference. Finally, induction treatments consisting of thymoglobulin, rituximab, corticosteroids, or a combination of these with or without Basiliximab were significantly associated with shorter duration of elevated CRP levels, compared to induction with Basiliximab only (**Table 3**).

In sub-group analysis, kidney transplant recipients who had at least one measurement of CRP levels above 50 mg/L and had received thymoglobulin (N = 37, 9.68%) had shorter duration of elevated CRP levels compared to kidney transplant recipients who did not receive Thymoglobulin (median duration of elevated CRP 2 days [IQR 1 to 3] vs 4 days [IQR 3 to 7], p<0.01). Similarly, recipients with at least one measurement of CRP above 50 mg/L who had received Rituximab (N = 41, 10.7%) had shorter duration of elevated CRP levels compared to kidney transplant recipients who did not receive Rituximab (median duration of elevated CRP 2 days [IQR 1 to 4] vs 4 days [IQR 3 to 7], p<0.01). The same was observed in recipients who received corticosteroid therapy and had at least one CRP measurement above 50 mg/L (N = 61, 15.96%), median duration of elevated CRP 2 days [IQR 1 to 4] vs 4 days [IQR 3 to 7], p<0.01).

In the second period, FOXP3 mRNA levels were not significantly associated with the count of days post-transplant in which CRP levels are above 50 mg/L (**Figure 5B**, **Table 3**). Due to the low incidence of CRP measurements above 50 mg/L in this period, we performed *post hoc* power calculation which revealed that this model was underpowered.

Prior kidney transplantation was the only covariate that was significantly associated with prolonged elevation of CRP in both periods. Compared to first-time transplant recipients (N = 442), recipients with prior kidney transplantations (N = 65) were treated more frequently with Thymoglobulin (p<0.01), Rituximab (p<0.01) and Corticosteroids (p<0.01). Compared to recipients with no prior transplantations, in samples collected at both the first day post-transplant and 29 days post-transplant, recipients with prior kidney transplantations had also lower levels of Total FOXP3, FOXP3fl and FOXP3d2 (**Supplemental Table 4**).

### 3.4 Association of FOXP3 mRNA levels with severity of inflammatory responses

To test the association between FOXP3 mRNA levels and the secondary outcome (maximal CRP level mg/L within each period), we performed linear regression analysis. For a better model fit, the outcome variables were converted to a logarithmic scale. We included recipient age, sex, prior kidney transplantation, donor type, type of induction therapy regiment, and number of comorbidities as potential confounding factors in the model.

**Figure 6:**
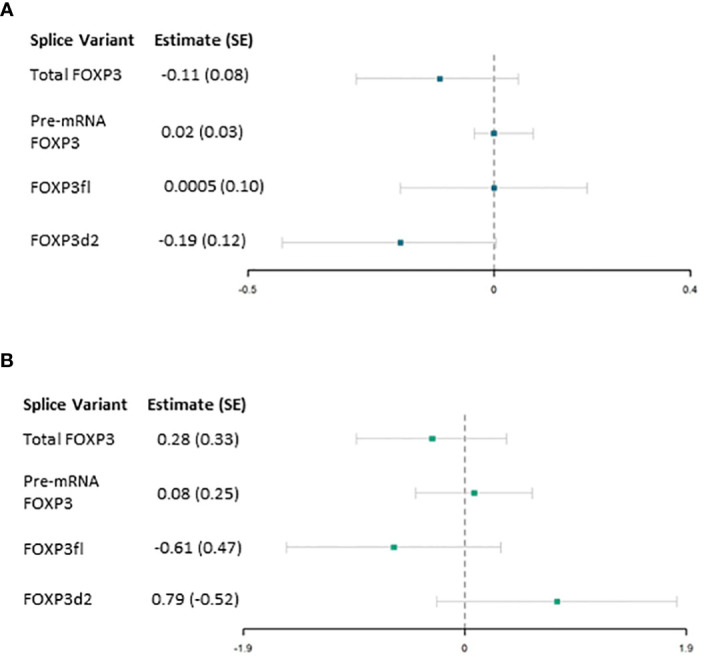
Results from Linear regression model . **(A)** association of forkhead box P3 (FOXP3) splice variants measured one day post-transplant and the secondary outcome (maximal level of C reactive protein mg/L) within the first period (2 to 29 days post-transplant) adjusted for confounding factors. **(B)** association of FOXP3 splice variants measured 29 days post-transplant and the primary outcome (maximal level of C reactive protein mg/L) within the second period (30 to 57 days post-transplant). SE, standard error.

No evidence of an association was found between FOXP3 mRNA levels and the secondary outcome within the first period (**Table 4**, **Figure 6A**). However, compared to females, males had a significant association with higher values of maximal CRP level by 0.11 (β 0.11, p = 0.04). Increasing number of comorbidities were significantly associated with higher values of maximal CRP (β 0.08, p = 0.03). Finally, compared to receiving Basliximab only, induction therapy with thymoglobulin and prednisolone, thymoglubuline, and prednisolone and rituximab were associated with lower values of maximal CRP (**Table 4**).

**Table 4:** Results of linear regression analysis, where the outcome variable is the maximal value of C reactive protein (mg/L) within each period.

	Outcome within 2 to 29 days post-transplant (N = 382)		Outcome within 30 to 57 days post-transplant (N = 69)	
Variable	Estimate ±standard error	P-value	Estimate ±standard error	P-value
Total FOXP3	-0.11 ± 0.08	0.19	-0.28 ± 0.33	0.40
Pre-mRNA FOXP3	0.02 ± 0.03	0.49	0.08 ± 0.25	0.75
FOXP3fl	0.0005 ± 0.10	0.99	-0.61 ± 0.47	0.20
FOXP3d2	-0.19 ± 0.12	0.12	0.79 ± 0.52	0.14
Age	0.003 ± 0.002	0.17	0.003 ± 0.007	0.65
Male sex	0.11 ± 0.05	0.04	-0.08 ± 0.20	0.68
Prior kidney transplantation	0.16 ± 0.08	0.06	-0.24 ± 0.26	0.36
Number of comorbidities	0.08 ± 0.04	0.03	0.05 ± 0.11	0.63
Donor type				
Deceased	Reference		Reference	
Living ABO-compatible	-0.006 ± 0.06	0.91	-0.10 ± 0.22	0.65
Living ABO-incompatible	-0.05 ± 0.24	0.81	-0.02 ± 0.68	0.97
Induction therapy				
Basiliximab	Reference		Reference	
Basiliximab and prednisolone	-0.25 ± 0.16	0.12	0.28 ± 0.51	0.57
Basiliximab, prednisolone and rituximab	-0.25 ± 0.26	0.33	-0.04 ± 0.78	0.95
Prednisolone and rituximab	-0.75 ± 0.37	0.04	–	–
Thymoglobulin	-0.45 ± 0.39	0.25	–	–
Thymoglobulin and prednisolone	-0.94 ± 0.22	< 0.01	0.0006 ± 0.53	0.99
Thymoglobulin, prednisolone and rituximab	-0.63 ± 0.16	< 0.01	0.19 ± 0.50	0.69

FOXP3, forkhead box P3. FOXP3fl, FOXP3 splice variant in which all exons are expressed. FOXP3d2, FOXP3 splice variant in which all exons but exon two are expressed. Total FOXP3, expression of both FOXP3fl and FOXP3d2 variants. Pre-mRNA: pre-mature RNA FOXP3 splice variants.

The model exploring the associations between FOXP3 mRNA levels with the secondary outcome variable in the second period showed no evidence of significant associations (**Table 4**, **Figure 6B**). The models were appropriate, and the assumption of linearity was fulfilled.

## 4 Discussion

Inflammatory episodes are a frequent cause of Kidney transplant recipient hospitalizations, morbidity, and mortality (24). Although it is comprehensible that treatment with several immunosuppressive agents or higher doses of immunosuppression may lead to a dysregulated or overreactive immune system, there is no simple biomarker that can quantify this relationship in individual kidney transplant recipients. In this prospective investigation, the results show that lower levels of Total FOXP3 mRNA in peripheral blood mononuclear cells are significantly associated with prolonged inflammatory response to stimuli in kidney transplant recipients.

CRP is a widely used and sensitive marker of inflammation (25). Regardless of the origin of stimuli, CRP is released by hepatocytes following stimulation by inflammatory cytokines, most notably IL-6 (26). Levels of CRP reflect the intensity of inflammatory stimuli (27–30), and given its short half-life in plasma of about 19 hours (30, 31), CRP is quickly cleared from the plasma when the inflammatory stimulus is eliminated. Thus, CRP is a reliable and sensitive marker that can be used to determine the duration of inflammatory responses. We chose a cut-off value of 50 mg/L to disregard CRP values that may indicate background chronic inflammatory processes, which tend to have lower levels of CRP elevation (16).

Human T regulatory cells express FOXP3 mRNA and proteins in three different isoforms, two of which, FOXP3fl and FOXP3d2, are essential for T regulatory cell differentiation, survival, and function (32, 33). T regulatory cells exert suppressive activities that modulate immune tolerance to auto- and allo-antigens (34, 35). T regulatory cells are also essential modulators during active inflammation. Initially during an inflammatory state, FOXP3 expression is downregulated by pro-inflammatory cytokines, such as IL-6, to enable an ample inflammatory response to stimuli. However, FOXP3 expression is promoted thereafter, through retinoic acid-dependent CCAAT/enhancer-binding protein activation, to protect nearby tissue from excessive inflammation (34). Our results show that measurements of lower Total FOXP3 mRNA in peripheral blood mononuclear cells, before episodes of inflammatory response, are associated with prolonged elevation of CRP levels in kidney transplant recipients. This suggests that low levels of Total FOXP3 mRNA expression in kidney transplant recipients may lead to a decrease in T regulatory cell suppression of active inflammation. More importantly, low levels of Total FOXP3 mRNA in peripheral blood mononuclear cells may highlight kidney transplant recipients who may react to inflammatory stimuli with a prolonged inflammatory response.

Kidney transplant recipients are treated with immunosuppressive medicines that target T cells, such as Basiliximab, Thymoglobulin, corticosteroids and calcineurin inhibitors (36, 37). These immunosuppressive medicines also target T regulatory cells and decrease FOXP3 expression, either by depletion of circulating T regulatory cells (3–7, 37) or by inhibition of FOXP3 expression (7, 9, 38–40). The decrease of FOXP3 expression is most notable early after transplantation, as FOXP3 expression increases transiently following transplantation (3, 7). This can also be seen in our *ad hoc* analysis, as FOXP3 levels decreased significantly following transplantation, and increased transiently thereafter. Furthermore, our results show that treatment regiments consisting of agents other than Basiliximab, or combinations of agents with Basiliximab, are associated with shorter durations of CRP elevation in kidney transplant recipients. We speculate that the association of multiple immunosuppressive drug treatments with shorter duration of CRP elevation may be explained by the effects of immunosuppressive medicines on the immune system. Multiple immunosuppressive induction agents, or depleting induction agents may impair the immune system’s ability to generate IL-6 and therefore also impair the subsequent CRP response. This is plausible as immunosuppressive medicines target IL-6 producing cells in the innate and adaptive immune functions (37, 41–43).

Of the examined co-factors, prior kidney transplantation was associated with prolonged CRP elevation in both periods. Recipients who have had prior kidney transplantation had significantly lower Total FOXP3, FOXP3fl and FOXP3d2 mRNA levels. The low levels of FOXP3 mRNA may indicate a lower number of circulating T regulatory cells or low expression of mature FOXP3 splice variants. A decrease in either circulating T regulatory cells, or FOXP3 expression in T regulatory cells, might have contributed to prolonged elevation of CRP during inflammatory responses. Furthermore, recipients who have had prior kidney transplantation were more frequently treated with Thymoglobulin, Rituximab and corticosteroids. Multiple transplantations also entail previous treatments with induction and maintenance therapy. The cumulative effects of multiple induction therapies, more frequent use Thymoglobulin and prior treatments with maintenance therapy might dysregulate FOXP3 expression and T regulatory cell actions during an inflammatory response and result in prolonged elevation of CRP levels.

Due to the low incidence of elevated CRP in the second period, the model exploring the association between FOXP3 mRNA levels measured 29 days post-transplant and the duration of CRP elevation was underpowered. This might explain why there was no evidence of association between FOXP3 mRNA levels measured 29 days post-transplant and duration of CRP elevation in the second period. However, FOXP3 mRNA levels were higher on day 29 compared to the first day post-transplant, which may have impacted the association. Congruently, we found no association between FOXP3 mRNA levels and maximal CRP levels within both periods. Due to the low incidence of elevated CRP in the second period, the model exploring FOXP3 mRNA levels measured 29 days post-transplant and maximal CRP within the second period is also likely underpowered. However, there was no evidence of an association between FOXP3 mRNA levels measured the first day post-transplant and maximal CRP in the first period, which suggests that baseline FOXP3 mRNA levels do not impact the magnitude of inflammatory responses, although Total FOXP3 mRNA levels may impact the duration of the inflammatory response.

To the best of our knowledge, this is the first study that explores the association between FOXP3 mRNA levels and prolonged CRP elevation during inflammatory responses in kidney transplant recipients. Although we found an association between Total FOXP3 mRNA levels and prolonged CRP elevation during inflammatory responses, this association was found in incident kidney transplant recipients. Thus, reproduction of our results is warranted in prevalent kidney transplant recipients. Finally, we attempted to control for confounding by including potential confounding factors in our multivariable regression models, but there is still a risk of confounding arising from unidentified factors.

In conclusion, low levels of peripheral blood Total FOXP3 mRNA transcript levels are associated with prolonged CRP elevation during active inflammatory responses in incident kidney transplant recipients. Total FOXP3 mRNA levels may reflect the effects of immunosuppressive medicines on T regulatory cell function, and the ability of T regulatory cells to limit inflammatory responses. Furthermore, Total FOXP3 mRNA levels may identify recipients with dysregulated T regulatory cell functions and guide clinical care.

## Data availability statement

The original contributions presented in the study are included in the article/**Supplementary Material**. Further inquiries can be directed to the corresponding authors.

## Ethics statement

The studies involving humans were approved by Den Videnskabsetiske Komite for Region Syddanmark, Project-ID: 20100098. The studies were conducted in accordance with the local legislation and institutional requirements. The participants provided their written informed consent to participate in this study.

## Author contributions

QS wrote the first draft, and participated in study conceptualization, development of methods, data curation and investigation. MT provided supervision and participated in study conceptualization, development of methods, and investigation. AM was responsible for statistical analysis and participated in methodology. All authors contributed to the article and approved the submitted version.
