# Supplementary material for: Lower levels of FOXP3 are associated with prolonged inflammatory responses in kidney transplant recipients
Source: Front Immunol. 2023 Sep 13;14:1252857. doi: 10.3389/fimmu.2023.1252857 (PMC10525697; doi:10.3389/fimmu.2023.1252857)
Supplement: Supplementary file 1 [file Table_1.docx]

**Supplemental material – table of content**

1 – Direct acyclic graph assumptions.

2 – Accessible donor data

3 – Comparison of pre- and post-transplant FOXP3 mRNA levels.

4 – subgroup analysis comparing immunosuppressive drug administration and FOXP3 splice variant levels in recipients who have had prior kidney transplantations.

**Supplemental Table 1** – Direct acyclic graph assumptions and sources.

| Potential confounding variable | Articles reporting association to FOXP3 | Articles reporting association to inflammatory response to stimuli |
| --- | --- | --- |
| Recipient age | (1-3) | (4-6) |
| Recipient sex | (7) | (4, 8, 9) |
| Donor type | (10) | (11) |
| Prior kidney transplantation | (10) | (12) |
| Immunosuppressive therapy | (13-19) | (20-22) |
| Number of comorbidities | (23-25) | (26-28) |

References:

1. Garg SK, Delaney C, Toubai T, Ghosh A, Reddy P, Banerjee R, et al. Aging is associated with increased regulatory T-cell function. Aging Cell. 2014;13(3):441-8.

2. Palatella M, Guillaume SM, Linterman MA, Huehn J. The dark side of Tregs during aging. Front Immunol. 2022;13:940705.

3. Thomas AL, Alarcon PC, Divanovic S, Chougnet CA, Hildeman DA, Moreno-Fernandez ME. Implications of Inflammatory States on Dysfunctional Immune Responses in Aging and Obesity. Front Aging. 2021;2:732414.

4. Tang Y, Liang P, Chen J, Fu S, Liu B, Feng M, et al. The baseline levels and risk factors for high-sensitive C-reactive protein in Chinese healthy population. Immunity & Ageing. 2018;15(1):21.

5. Wyczalkowska-Tomasik A, Czarkowska-Paczek B, Zielenkiewicz M, Paczek L. Inflammatory Markers Change with Age, but do not Fall Beyond Reported Normal Ranges. Arch Immunol Ther Exp (Warsz). 2016;64(3):249-54.

6. van Vught LA, Endeman H, Meijvis SC, Zwinderman AH, Scicluna BP, Biesma DH, et al. The effect of age on the systemic inflammatory response in patients with community-acquired pneumonia. Clinical Microbiology and Infection. 2014;20(11):1183-8.

7. Singh RP, Bischoff DS. Sex Hormones and Gender Influence the Expression of Markers of Regulatory T Cells in SLE Patients. Front Immunol. 2021;12:619268.

8. Corcoran MP, Meydani M, Lichtenstein AH, Schaefer EJ, Dillard A, Lamon-Fava S. Sex hormone modulation of proinflammatory cytokine and C-reactive protein expression in macrophages from older men and postmenopausal women. Journal of Endocrinology. 2010;206(2):217-24.

9. Clark DO, Unroe KT, Xu H, Keith NR, Callahan CM, Tu W. Sex and Race Differences in the Relationship between Obesity and C-Reactive Protein. Ethn Dis. 2016;26(2):197-204.

10. Saleh QW NS, Rasmussen M, Tepel M. Thymoglobulin reduces regulatory T cell specific forkhead box P3 (FOXP3) transcripts in kidney transplant recipients. Nordiske Nyredage; 05/05/2022; Odense. Odense2022.

11. Kim JS, Jeong KH, Lee DW, Lee SY, Lee SH, Yang J, et al. Epidemiology, risk factors, and clinical impact of early post-transplant infection in older kidney transplant recipients: the Korean organ transplantation registry study. BMC Geriatrics. 2020;20(1):519.

12. Alangaden GJ, Thyagarajan R, Gruber SA, Morawski K, Garnick J, El-Amm JM, et al. Infectious complications after kidney transplantation: current epidemiology and associated risk factors. Clinical Transplantation. 2006;20(4):401-9.

13. Bouvy AP, Klepper M, Kho MM, Boer K, Betjes MG, Weimar W, et al. The impact of induction therapy on the homeostasis and function of regulatory T cells in kidney transplant patients. Nephrol Dial Transplant. 2014;29(8):1587-97.

14. Krystufkova E, Sekerkova A, Striz I, Brabcova I, Girmanova E, Viklicky O. Regulatory T cells in kidney transplant recipients: the effect of induction immunosuppression therapy. Nephrology Dialysis Transplantation. 2011;27(6):2576-82.

15. López-Abente J, Martínez-Bonet M, Bernaldo-de-Quirós E, Camino M, Gil N, Panadero E, et al. Basiliximab impairs regulatory T cell (TREG) function and could affect the short-term graft acceptance in children with heart transplantation. Sci Rep. 2021;11(1):827.

16. Tang Q, Leung J, Melli K, Lay K, Chuu EL, Liu W, et al. Altered balance between effector T cells and FOXP3+ HELIOS+ regulatory T cells after thymoglobulin induction in kidney transplant recipients. Transpl Int. 2012;25(12):1257-67.

17. Krepsova E, Tycova I, Sekerkova A, Wohlfahrt P, Hruba P, Striz I, et al. Effect of induction therapy on the expression of molecular markers associated with rejection and tolerance. BMC Nephrol. 2015;16:146.

18. Kim SH, Oh EJ, Ghee JY, Song HK, Han DH, Yoon HE, et al. Clinical significance of monitoring circulating CD4+CD25+ regulatory T cells in kidney transplantation during the early posttransplant period. J Korean Med Sci. 2009;24 Suppl(Suppl 1):S135-42.

19. Karagiannidis C, Akdis M, Holopainen P, Woolley NJ, Hense G, Rückert B, et al. Glucocorticoids upregulate FOXP3 expression and regulatory T cells in asthma. J Allergy Clin Immunol. 2004;114(6):1425-33.

20. Fishman JA, Costa SF, Alexander BD. Infection in Kidney Transplant Recipients. Kidney Transplantation - Principles and Practice. 2019:517-38.

21. Roberts MB, Fishman JA. Immunosuppressive Agents and Infectious Risk in Transplantation: Managing the "Net State of Immunosuppression". Clin Infect Dis. 2021;73(7):e1302-e17.

22. Fishman JA. Infection in Organ Transplantation. Am J Transplant. 2017;17(4):856-79.

23. Qiao YC, Shen J, He L, Hong XZ, Tian F, Pan YH, et al. Changes of Regulatory T Cells and of Proinflammatory and Immunosuppressive Cytokines in Patients with Type 2 Diabetes Mellitus: A Systematic Review and Meta-Analysis. J Diabetes Res. 2016;2016:3694957.

24. Joly A-L, Seitz C, Liu S, Kuznetsov NV, Gertow K, Westerberg LS, et al. Alternative Splicing of FOXP3 Controls Regulatory T Cell Effector Functions and Is Associated With Human Atherosclerotic Plaque Stability. Circulation Research. 2018;122(10):1385-94.

25. Katsuki M, Hirooka Y, Kishi T, Sunagawa K. Decreased proportion of Foxp3+ CD4+ regulatory T cells contributes to the development of hypertension in genetically hypertensive rats. J Hypertens. 2015;33(4):773-83; discussion 83.

26. Chang C-H, Wang J-L, Wu L-C, Chuang L-M, Lin H-H. Diabetes, Glycemic Control, and Risk of Infection Morbidity and Mortality: A Cohort Study. Open Forum Infectious Diseases. 2019;6(10).

27. Bae S, Kim SR, Kim M-N, Shim WJ, Park S-M. Impact of cardiovascular disease and risk factors on fatal outcomes in patients with COVID-19 according to age: a systematic review and meta-analysis. Heart. 2021;107(5):373-80.

28. Santoro A, Franceschini E, Meschiari M, Menozzi M, Zona S, Venturelli C, et al. Epidemiology and Risk Factors Associated With Mortality in Consecutive Patients With Bacterial Bloodstream Infection: Impact of MDR and XDR Bacteria. Open Forum Infect Dis. 2020;7(11):ofaa461.

**Supplemental Table 2 –** Accessible Donor data.

Table footnotes: Continuous values are reported as median [interquartile range], and categorical values are reported as number (percent).

| Donor Characteristics | Statistic |
| --- | --- |
| Age (years), N = 336 | 55 [46 to 64] |
| Male sex, N (%), N = 343 | 111 (45%) |
| Cold ischemic time (minutes), N= 282 | 780 [585 to 1050] |

**Supplemental Table 3 –** Logarithmic values of normalized forkhead box P3 (FOXP3) splice variant levels in kidney transplant recipients according to day of sample collection.

Table footnotes: Data are presented as median [interquartile range]. Comparisons were performed with Wilcoxon rank sum test. Tx: transplantation. Total FOXP3: detects the two most abundant splice variants, FOXP3fl and FOXP3d2. Pre-mRNA FOXP3: detects pre-mRNA that contain introns. FOXP3fl: detects mature FOXP3 mRNA that includes all exons. FOXP3d2: detects mature FOXP3 mRNA that skip exon 2.

| Logarithmic value of | Pre-Tx | First day post-TX | 29 days post-Tx | Comparison of pre-TX and first day post-Tx | Comparison of first day and day 29 post-Tx | Comparison of pre-TX and day 29 post-Tx |
| --- | --- | --- | --- | --- | --- | --- |
| Total FOXP3 | -3.31 [-3.52 to -3.10] | -3.65 [-3.96 to -3.37] | -3.58 [-3.97 to -3.33] | P < 0.01 | P = 0.05 | P < 0.01 |
| Pre-mRNA FOXP3 | -4.29 [-4.49 to -4.10] | -4.46 [-4.68 to -4.18] | -4.36 [-4.62 to -4.13] | P < 0.01 | P < 0.01 | P = 0.05 |
| FOXP3fl | -3.66 [-3.88 to -3.41] | -4.00 [-4.33 to -3.73] | -3.89 [-4.24 to -3.63] | P < 0.01 | P < 0.01 | P < 0.01 |
| FOXP3d2 | -3.47 [-3.63 to -3.32] | -3.75 [-3.98 to -3.52] | -3.72 [-3.97 to -3.45] | P < 0.01 | P = 0.05 | P < 0.01 |

**Supplemental Table 4** – Subgroup analysis comparing immunosuppressive drug administration and FOXP3 splice variant levels in recipients who have had prior kidney transplantations and for first-time transplant recipients.

| Variable | First-time transplant recipient | Prior kidney transplant recipient | P-value |
| --- | --- | --- | --- |
| Basiliximab, N (%) | 383 (87 %) | 37 (57 %) | < 0.01 |
| Rituximab, N (%) | 67 (15 %) | 27 (42 %) | < 0.01 |
| Thymoglobulin, N (%) | 51 (12 %) | 28 (43 %) | < 0.01 |
| Corticosteroids, N (%) | 102 (23 %) | 36 (55 %) | < 0.01 |
| Logarithmic values of FOXP3 levels first post-transplant day  Total FOXP3  Pre-mRNA FOXP3  FOXP3fl  FOXP3d2 | -3.66 [-3.89 to -3.39]  -4.43 [-4.69 to -4.12]  -3.97 [-4.27 to -3.75]  -3.74 [-3.98 to -3.53] | -4.03 [-4.57 to -3.68]  -4.41 [-4.76 to -4.23]  -4.25 [-4.80 to -3.95]  -3.99 [-4.47 to -3.61] | < 0.01  0.40  < 0.01  < 0.01 |
| Logarithmic values of FOXP3 levels 29 days post-transplant  Total FOXP3  Pre-mRNA FOXP3  FOXP3fl  FOXP3d2 | -3.57 [-3.84 to -3.30]  -4.34 [-4.62 to -4.11]  -3.85 [-4.16 to -3.63]  -3.66 [-3.95 to -3.44] | -3.83 [-4.47 to -3.52]  -4.44 [-4.70 to -4.08]  -4.14 [-4.52 to -3.88]  -3.89 [-4.27 to -3.69] | < 0.01  0.40  < 0.01  < 0.01 |
